# Test of a biobehavioral model linking weight suppression to binge-eating severity via leptin and glucagon-like peptide 1 in bulimia nervosa and related syndromes in women

**DOI:** 10.1017/S0033291725100871

**Published:** 2025-06-27

**Authors:** Pamela K. Keel, Lindsay P. Bodell, Jonathan Appelbaum, Diana L. Williams

**Affiliations:** 1 Department of Psychology, Florida State University, Tallahassee, FL, USA; 2 Department of Psychology, Western University, London, Ontario, Canada; 3 College of Medicine, Florida State University, Tallahassee, FL, USA; 4 Kravis Department of Integrated Sciences, Claremont McKenna College, Claremont, CA, USA

**Keywords:** binge eating, bulimia nervosa, eating disorders, GLP-1, glucagon-like peptide 1, leptin, reward, satiation, weight suppression

## Abstract

**Background:**

Weight suppression represents the difference between highest and current body weight and predicts maintenance of bulimia nervosa and related syndromes (BN-S). This study tested a biobehavioral model of binge-eating severity in which greater weight suppression links to reduced leptin, which links to reduced glucagon-like peptide 1 (GLP-1) release, which links to both decreased reward satiation and increased reward valuation, which link, respectively, to excessive food intake and loss of control while eating – the defining features of DSM-5 binge-eating episodes.

**Methods:**

Women (*N* = 399) who met DSM-5 criteria for bulimia nervosa or another eating disorder with binge eating (*n* = 321) or had no lifetime eating disorder symptoms (*n* = 78) participated in a multi-visit protocol, including structured clinical interviews, height, weight, weight history, percent body fat, fasting leptin, post-prandial GLP-1 response to a fixed meal, and self-report and behavioral assessments of food reward satiation (*ad lib* meal) and food and nonfood reward valuation (progressive ratio tasks).

**Results:**

A structural equation model (SEM) demonstrated excellent fit to data with significant pathways from greater weight suppression to lower leptin, to blunted GLP-1 response, to lower reward satiation, to larger eating/binge-eating episode size, with significant indirect paths through leptin, GLP-1, and reward satiation. SEM with paths via reward valuation to loss of control eating demonstrated inadequate fit.

**Conclusions:**

Findings specifically link reduced GLP-1 response to severity of binge-episode size and support weight history assessment in eating disorders, DSM-5 over ICD-11 criteria for binge eating, and may inform future clinical trials of GLP-1 agonists for BN-S.

## Introduction

Bulimia nervosa (BN) and related syndromes (BN-S), including anorexia nervosa binge-purge subtype (ANbp), binge-eating disorder (BED), and other specified feeding or eating disorder (OSFED), are severe and life-threatening psychiatric disorders characterized by binge eating that disproportionally impact young women (Crow et al., 2009; Udo & Grilo, 2019, 2022). Fluoxetine is the only FDA-approved treatment for BN, while lisdexamfetamine is the only FDA-approved BED medication, and both treatments demonstrate variable efficacy, and the FDA has approved no medication for AN (Fornaro et al., 2023). Reducing BN-S morbidity and mortality requires better identification of underlying mechanisms for their core symptom – binge eating. Two features define binge eating in the DSM-5 – consuming large amounts of food within a limited period and experiencing a loss of control (LOC) while eating. We developed a biobehavioral model of binge-eating severity and maintenance from evidence that a greater highest-to-current adult weight difference, termed weight suppression (WS) (Lowe, 1993), predicted worse treatment response and long-term outcomes (Keel et al., 2019). The model posited that weight suppression triggered changes in Research Domain Criteria (RDoC) reward constructs corresponding to binge-eating’s defining features. Weight suppression decreased the ability to achieve a state of satisfaction or completion when freely consuming a reward, termed reward satiation, and this contributed to excessive food intake. Simultaneously, weight suppression increased the drive to consume a reward, termed reward valuation, contributing to LOC. Reduced leptin and postprandial glucagon-like peptide 1 (GLP-1) response play central roles in the biobehavioral model, with blunted GLP-1 response as a posited mechanism and potential future treatment target for BN-S (Keel et al., 2019).

In support of this model, greater weight suppression predicts BN-S severity (Butryn, Juarascio, & Lowe, 2011; Keel & Heatherton, 2010; Lowe, Thomas, Safer, & Butryn, 2007) and maintenance (Butryn, Lowe, Safer, & Agras, 2006; Keel & Heatherton, 2010; Lowe et al., 2011), controlling for age, body mass index (BMI), body image disturbance, and dietary restraint (Butryn, Juarascio, & Lowe, 2011; Keel & Heatherton, 2010). Furthermore, greater weight suppression significantly correlates with lower leptin, controlling for BMI (Bodell & Keel, 2015; Keel et al., 2017) and percent body fat (Bodell & Keel, 2015). Leptin crosses the blood–brain barrier, modulating food intake via hypothalamic and reward circuit activation (Stefanakis et al., 2024), and acts via peripheral meal-related signals (Woodward, Gribble, Reimann, & Lewis, 2022), including potently stimulating postprandial GLP-1 release in rodents (Anini & Brubaker, 2003; Williams & Elmquist, 2012). Experimental manipulation of GLP-1 in animals impacts food reward satiation measured via *ad lib* food intake and food and nonfood reward valuation measured in progressive ratio tasks (Woodward et al., 2022). In an *ad lib* task, food is freely available, and intake terminates when the subject is sated. In contrast, subjects must exert increasing levels of effort in a progressive ratio task to access and consume food, and intake terminates when the effort required exceeds the food’s reinforcing value. Moreover, BN is characterized by lower leptin (Bodell & Keel, 2015; Cassioli et al., 2024), reduced postprandial GLP-1 response (Balantekin, Kretz, & Mietlicki-Baase, 2024; Dossat et al., 2015), greater *ad lib* food intake (Geliebter et al., 1992; Hadigan et al., 1992; Keel, Haedt-Matt et al., 2018), and higher motivation for food and nonfood rewards on progressive ratio tasks (Bodell & Keel, 2015; Bulik & Brinded, 1994; Schebendach, Broft, Foltin, & Walsh, 2013) compared to controls. Our lab extended evidence of blunted GLP-1 response (Dossat et al., 2015) and decreased satiation (Keel, Haedt-Matt et al., 2018) in women with BN compared those with purging disorder – a condition characterized by purging in the absence of binge-eating episodes (Keel, Haedt, & Edler, 2005). This last finding addresses model specificity to binge-eating but does not address whether the model generalizes to other eating disorders characterized by binge eating. Finally, we found that leptin statistically mediated the association between weight suppression and reported duration of illness in BN-S cross-sectionally (Keel et al., 2017) but did not have measures of GLP-1 function or behavioral reward valuation or satiation tasks to test the full model.

This study aimed to test *a priori* hypotheses that individual differences in weight suppression link to reduced leptin, which link to reduced GLP-1 release, which link to both decreased reward satiation and increased reward valuation, which link, respectively, to eating/binge-eating episode size and LOC frequency (Keel et al., 2019). We also sought to determine whether biobehavioral measures statistically mediated associations between weight suppression and BN-S severity outcomes. Finally, analyses tested whether the model accounted for variance in global eating disorder severity.

## Methods

A complete report of the protocol and sample is provided in an open-access article (Keel et al., 2025).

### Participants

To measure RDoC constructs dimensionally from a state of health to disease (Cuthbert & Insel, 2013), participants (*N* = 399) were recruited from the community with no eating disorder history (*n* = 78) or a current BN-S (*n* = 321), including DSM-5 anorexia nervosa-binge-purge subtype (ANbp; *n* = 8), BN (*n* = 156), binge-eating disorder (BED; *n* = 4), and other specified feeding or eating disorder (OSFED; *n* = 11 atypical AN; *n* = 136 BN low frequency/duration; *n* = 4 BED low frequency/duration; *n* = 5 other/unspecified). All BN-S participants were required to endorse objectively large binge episodes, defined by experiencing LOC and consuming >1,000 kcal within 2 h and exceeding what most people would consume under similar circumstances. The >1,000 kcal threshold distinguishes between individuals with and without binge-eating episodes in feeding lab studies (Mitchell et al., 1998) and has been validated by distinct biological and behavioral correlates in comparisons of BN to purging disorder (Keel et al., 2007; Keel, Eckel et al., 2018; Keel, Haedt-Matt et al., 2018). For a current DSM-5 OSFED diagnosis, minimum behavioral symptom frequency was set to a combined average of once weekly for objective binge episodes, subjective binge episodes, purging, and nonpurging inappropriate compensatory behaviors, to align with the minimum behavioral symptom frequency for a DSM-5 diagnosis of BED. Inclusion criteria were being female, based on biological sex assigned at birth, aged 18 and 35 years, BMI between 16 to 35 kg/m^2^, liking reward stimuli, no conditions/medications that influence weight, appetite, or ability to complete the protocol. Except for hormonal contraception and stable SSRI dose, which were permitted, participants were free of all medications and substances for biobehavioral assessments. The BMI range included BN-S across DSM-5 diagnoses, extending from ANbp to BED. Inclusion/exclusion criteria were evaluated during initial telephone screens probing whether participants liked playing computer games, frozen yogurt, and M&Ms®, medications, medical conditions, and food allergies.

Race and ethnicity were collected via self-report according to the National Institutes of Health required categories. Table 1 includes variable ranges and reliability. Subjects provided written informed consent after receiving a complete description of the study.

**Table 1.** Measurement and sample characteristics on variables included in structural equation models

Variables included in SEM analyses	Range	Reliability	Sample characteristics		
			N	Mean/*n* (yes)	SD/% (yes)
1. Weight suppression (WS), %	0–36.78^a^	0.95^b^	399	6.41	6.27
2. Leptin, ng/mL	2.70–133.98^a^	0.89^b^	340	29.52	21.65
3. Glucagon-like peptide 1 (GLP–1) total AUC, pM x min	137.20–3537.08^a^	0.70^b^	292	1376.59	624.80
4. Ad lib intake, grams	6.70–972.30^a^	NA	287	246.04	148.35
5. VAS hunger (reverse scored), AUC, mm x min	647.50–4600.50^a^	NA	298	2972.45	765.93
6. VAS fullness, AUC, mm x min	0–4352.50^a^	NA	298	2374.18	765.96
7. VAS satiation, AUC, mm x min	0–4168.00^a^	NA	297	1940.66	839.47
8. Reward Valuation – Effort (RV-E) food reward, breakpoint	50–1850	0.85^b^	255	767.65	469.84
9. RV-E game reward in fasted state, breakpoint	50–1850	0.91^b^	347	844.81	478.53
10. RV-E game reward in fed state, breakpoint	50–1850	NA	291	609.45	406.34
11. VAS want food reward before task, mm	0–100	0.78^b^	255	66.42	27.64
12. VAS want game reward before fasted task, mm	0–100	0.69^b^	344	45.91	23.62
13. VAS want game reward before fed task, mm	0–100	NA	294	34.63	26.99
14. Eating/binge-eating episode size, kcal	472.5–14010^a^	0.82^c^	399	2630.98	1570.07
15. Loss of control (LOC) frequency, number in 12 weeks	0–232^a^	>0.99^c^	399	35.11	40.25
16. Eating Disorder Examination (EDE) score	0–6	>0.99^c^	399	2.57	1.50
17. Age, years	18–35	NA	399	20.26	2.58
18. Body mass index (BMI), kg/m^2^	16.5–35	0.95^b^	399	24.50	4.25
19. Percent body fat, %	7.60–49.30^a^	0.94^b^	399	30.78	7.96
20. Hormonal contraceptive (HC) use (0 = no; 1 = yes)	NA	NA	399	183	45.9
21. SSRI Use (0 = no; 1 = yes)	NA	NA	399	38	9.5
22. Current DSM–5 depressive disorder diagnosis (0 = no; 1 = yes)	NA	0.95^d^	399	121	30.3
23. Current DSM–5 substance use disorder diagnosis (0 = no; 1 = yes)	NA	0.83^d^	399	136	34.0
24. Enrolled after COVID–19 pandemic onset (0 = no; 1 = yes)	NA	NA	399	98	24.6

Abbreviations: AUC = area under curve; SSRI = selective serotonin reuptake inhibitor; VAS = visual analogue scale.

a Observed range reported.

b Test–retest reliability/stability (r-value) was calculated from fasted measures on separate days.

c Intraclass correlation for interrater reliability of random selection of 16.7% of interviews. Internal consistency reliability for EDE Global Score was α = .90.

d Kappa for interrater reliability of random selection of 16.7% of interviews.

### Study design and setting

Cross-sectional data collection began on November 2016 in a clinical research lab at Florida State University (FSU), paused March 2020 to February 2021, and ended December 2022 with prior approval from the FSU Institutional Review Board for all methods and adjustments made in response to the onset of the COVID-19 pandemic in March of 2020.

**Figure 1.**
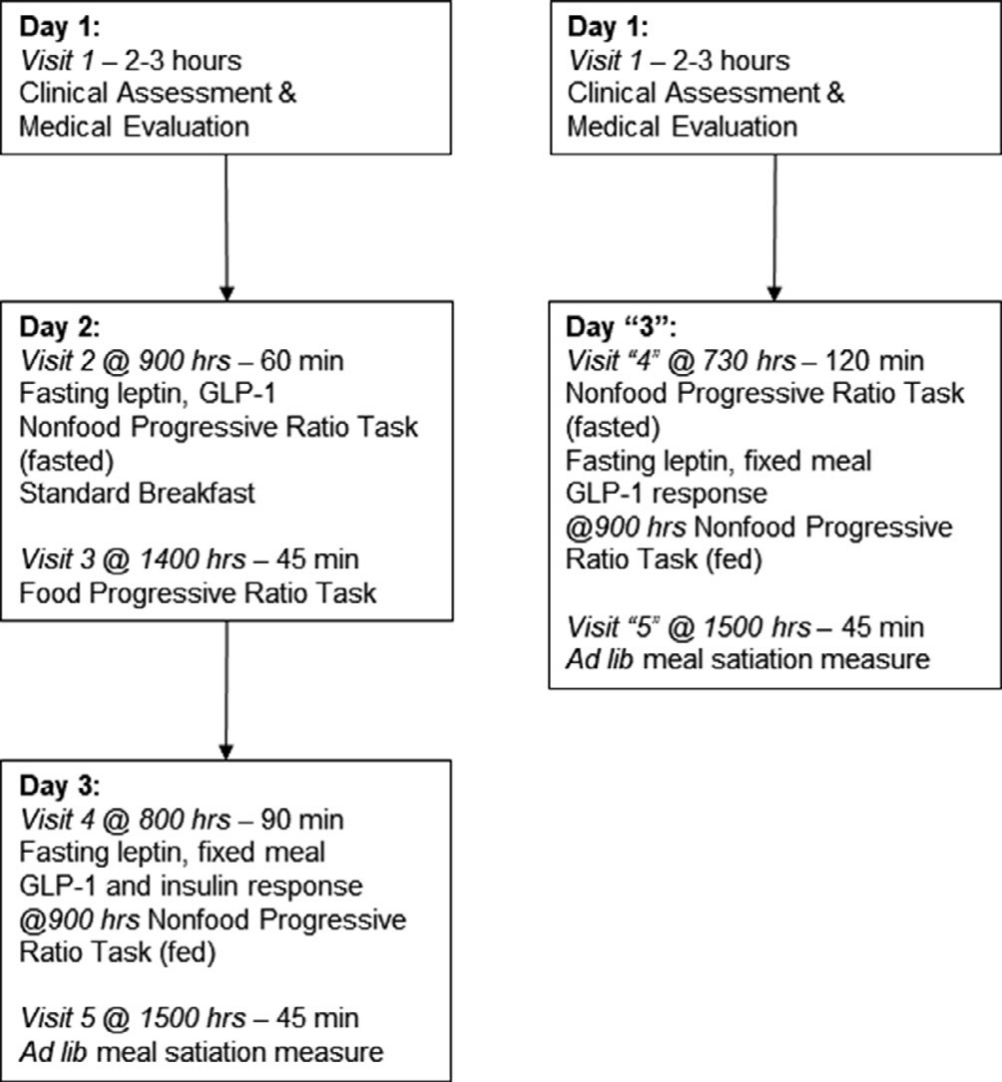
Abbreviations: GLP-1 = glucagon-like peptide 1.

Figure 1 depicts the protocol and pandemic-related adjustments (full details reported elsewhere (Keel et al., 2025)). The first visit established eligibility and clinical features via semi-structured clinical interviews, questionnaires, and medical evaluations, including weight suppression and BMI. Participants played Angry Birds™ for 1 min, tasted the frozen yogurt, and M&Ms® and rated how much they ‘liked’ each stimulus on Visual Analogue Scales from 0 = ‘Not at All’ to 100 = ‘Extremely’ to confirm eligibility. Participants also consumed the full fixed meal during this visit, replicating our prior methods (Keel et al., 2007; Keel, Eckel et al., 2018). Subsequent visits provided fasting leptin, postprandial GLP-1 response, and self-reported reward satiation to the fixed meal. *Ad lib* meal intake captured behavioral food reward satiation. Progressive ratio tasks measured behavioral reward valuation-effort for food in a semi-fasted state pre-pandemic (*N* = 255) and for a nonfood reward in fasted and fed states with pre-task self-reported reward valuation. Participants were compensated $75 for Day 1, $50 for Day 2, $100 for Day 3, and a $35 bonus for completing visits without rescheduling. Post-COVID-19 enrolled participants were paid $75 for Day 1, $110 for Day ‘3’, and a $15 bonus.

### Measurement

*WS, BMI and percent body fat.* Current weight and height were measured to calculate BMI (kg/m^2^) each day using a digital scale and stadiometer. Percent body fat was measured using bioelectrical impedance analysis (Tanita Corporation of America, Arlington Heights, IL), which demonstrates high correlations (r = 0.88–0.94) with dual-energy X-ray absorptiometry (Boneva-Asiova & Boyanov, 2008; Sun et al., 2005). Highest BMI was calculated from self-reported highest adult weight at current height, not including pregnancy. Based on theory and empirical evidence (Lowe, Piers, & Benson, 2018; Schaumberg et al., 2016), weight suppression was defined as percent loss from highest to current BMI; ([highest BMI – current BMI]/highest BMI) × 100.

*Leptin and GLP-1.* Leptin and GLP-1 were measured in the morning after an overnight fast. Blood was drawn once for fasting leptin and before (−5 min) and repeatedly after (+5, +15, and + 30 min) the fixed meal for GLP-1 response, which peaks 15–20 min postprandially (Dossat et al., 2015). We focused on fasting leptin as a direct correlate of body composition and potential consequence of weight suppression. We focused on GLP-1 area under the curve (AUC) as a meal-related response posited to influence both reward satiation and reward valuation. Enzyme linked immunosorbent assays (MilliporeSigma, Burlington, MA) of plasma determined leptin (EZHL-80SK), active GLP-1 (EZGLPHS-35 K), and total GLP-1 (EZGLP1T-36 K). Assays proportionally balanced inclusion of samples from control and BN-S participants. Dr. Williams reviewed results to identify out-of-range values and acceptable CVs blind to clinical data. When Dr. Williams flagged unreliable values, back-up plasma samples were included in subsequent assays. Mean intra−/inter-assay CVs for leptin (4.3%/9.0%), active GLP-1 (7.4%/9.5%), and total GLP-1 (2.9%/10.3%) were acceptable.

*Fixed meal.* 660 grams of Ensure Plus®; 900 kcal: 30% fat, 15% protein, and 55% carbohydrate were consumed from −5 min to 0 min after overnight fast (Keel et al., 2007).

*Ad lib meal.* 1420 grams (1.5 quarts) of vanilla frozen yogurt was served in a private room with printed and recorded instructions to eat until full/satiated (Keel, Haedt-Matt et al., 2018). The use of frozen yogurt in a single-item *ad lib* meal replicated methods distinguishing women with BN from controls (Wolfe, Metzger, & Jimerson, 2002) and women with purging disorder (Keel, Haedt-Matt et al., 2018). Grams consumed provided behavioral food reward satiation.

*Progressive ratio tasks.* Tasks were developed and validated in prior studies to measure reward valuation-effort of food (Bodell & Keel, 2015) and nonfood rewards (Keel et al., 2022) used in the current study. As measures of absolute reinforcing value, participants worked to earn one type of reward. Briefly, participants were told they could earn M&Ms® on the food task [or Angry Birds™ on the game task] by pressing a computer key, that the task consisted of 10 trials, and that they would receive and consume 10 M&M’s® [or play Angry Birds™ for 1 min] after each trial. Participants were instructed to work for the amount they wanted, that they could press the key as little or as much as they chose, could stop at any time, and there were no right or wrong answers. Replicating prior work (Schebendach et al., 2013), the first trial required 50 presses, and increased by 200 presses (250 for trial 2, 450 for trial 3… 1850 for trial 10). After each trial was completed, the dispenser distributed 10 M&M’s® [or the screen opened Angry Birds™ for 1 min] for immediate consumption. Participants were left alone and asked to notify the researcher when they completed all 10 trials or decided to stop. Breakpoint is the number of key presses in the last completed trial, representing reinforcing value for a reward (Epstein, Leddy, Temple, & Faith, 2007). A latent variable for reward valuation-effort was calculated from progressive ratio tasks’ breakpoints.

*Visual Analogue Scales (VAS)* captured momentary ratings on a 100 mm line from ‘None/Not at all’ to ‘Extreme/Extremely’ during behavioral tasks (Keel et al., 2007). A latent self-reported reward satiation variable included AUCs of ‘satiation/satisfied’, ‘hunger’ (reverse scored), and ‘fullness’ ratings from −5 to +30 min over the fixed meal. A latent self-reported reward valuation variable included pre-progressive ratio task ratings of how much the participant ‘wanted’ the reward (game play/M&Ms®).

The *Eating Disorders Examination 17.0D* (Fairbum, Cooper, & O’Connor, 2014) established: (1) eating/binge-eating episode size, (2) LOC frequency, and (3) global eating disorder severity. The EDE captures the amount of food consumed separately from LOC during eating episodes. To capture largest eating episode, participants were asked to describe the largest amount they had eaten within a limited period of time over the prior 12 weeks, replicating methods distinguishing purging disorder from BN and validated by distinct physiological (Dossat et al., 2015; Keel et al., 2007; Keel, Eckel et al., 2018) and behavioral indicators (Keel, Haedt-Matt et al., 2018). Food models, measuring cups, and plates and bowls of assorted sizes were offered to aid participants’ reports on the type and amount of each food eaten. Calories were obtained from on-line nutritional information provided by restaurants and food companies and the on-line nutritional database calorieking.com. EDE (Fairbum et al., 2014) algorithms established current DSM-5 eating disorder diagnoses and symptom features over 12 weeks, including LOC frequency as the sum of objective and subjective bulimic episodes, eating/binge-eating episode size as the largest number of kCal consumed in 2 h, and purging frequency as the sum of episodes of self-induced vomiting, laxative, and diuretic misuse. EDE interview global score measured eating disorder severity.

The *Structured Clinical Interview for DSM-5* (SCID-5) (First, Williams, Karg, & Spitzer, 2014) captured lifetime eating disorder diagnoses, with excellent interrater reliability, AN κ = 0.93, BN κ = 0.92, BED κ = 0.88, and OSFED κ = 0.92, and lifetime and current diagnoses of related disorders (see Table 1).

### Statistical analyses

Analyses were performed using SPSS (version 29; IBM) or MPLUS (version 8.7). Variable distributions were inspected for outliers and normality. Repeated fasting measures of weight suppression, leptin, BMI, and percent body fat were averaged. Square root transformation of *ad lib* intake, eating/binge-eating episode size, and LOC frequency corrected for skew/kurtosis (Hahs-Vaughn & Lomax, 2020). To control for inter-assay variability, standardized residuals from models predicting leptin and GLP-1 values from assay kit were saved and used in analyses. Bivariate correlations preceded structural equation modeling (SEM) (see Table S1 in the online supplement for details).

**Figure 2.**
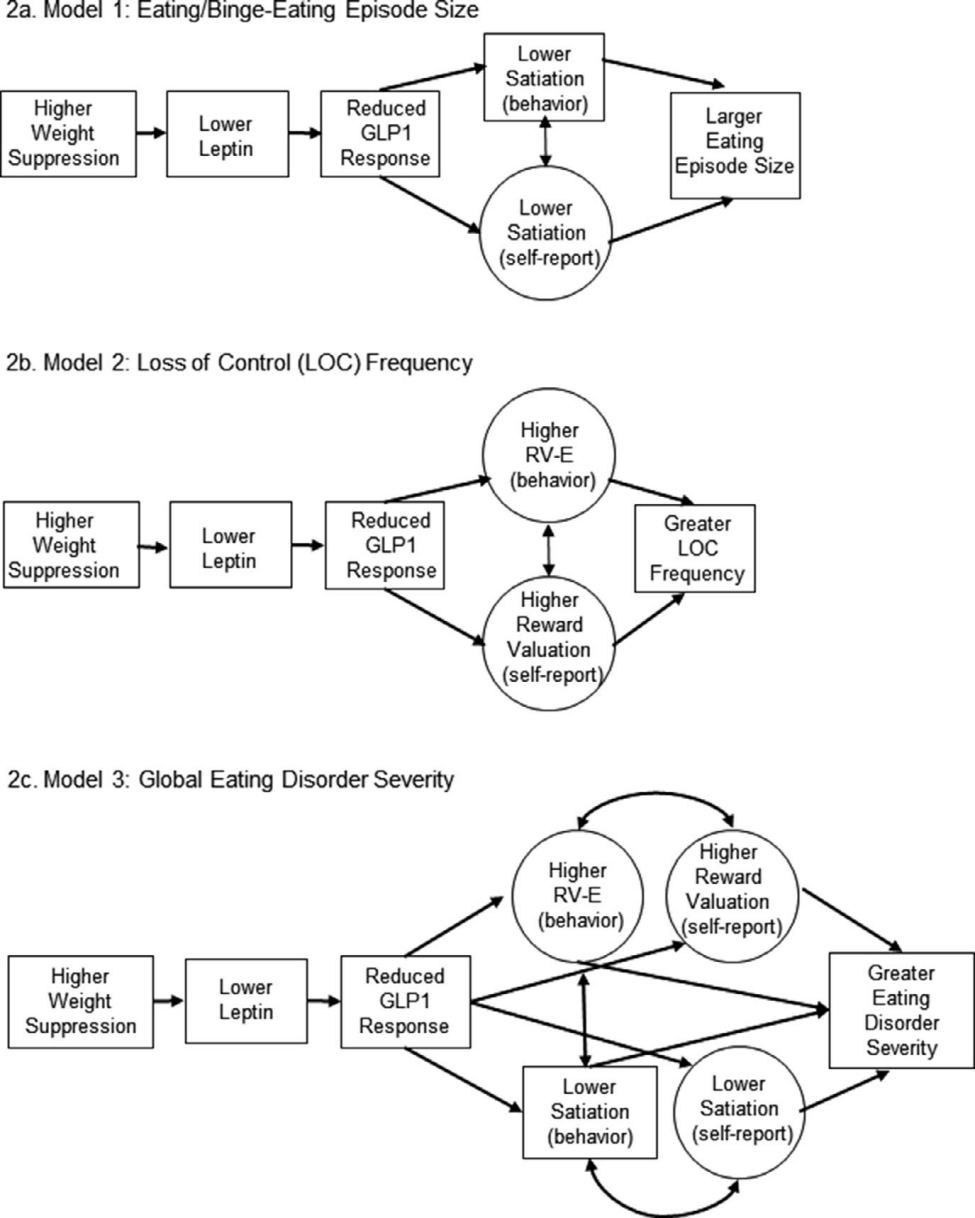
Abbreviations: GLP-1 = glucagon-like peptide 1; RV = reward valuation; RV-E = reward valuation-effort Note: Circles represent latent variables and squares represent observed variables. The latent variable for satiation (self-report) consisted of three indicators: Area under the curve visual analog ratings for fullness, satiation, and hunger (reversed scored) during the fixed meal. Lower satiation (behavior) is represented by greater food intake during the *ad lib* meal. The latent variable for reward valuation-effort (behavior) consisted of three indicators: progressive ratio food task breakpoint; progressive ratio game task [fasted state] breakpoint; progressive ratio game task [fed state] breakpoint. The latent variable for reward valuation (self-report) included three indicators: visual analog scale ratings for how much participants ‘want’ the reward for which they were about to work administered just prior to each progressive ratio task. Weight suppression was calculated as percent of body mass index (BMI) loss from highest adult BMI and current BMI. Leptin represents standardized residuals of average fasting leptin values across two separate days, controlling for assay in which samples were run. GLP1-response represents standardized residuals for GLP1-total response, controlling for assay in which samples were run.

SEM indicated model fit with hypothesized associations between weight suppression and BN-S severity via reduced leptin, blunted GLP1-response, and reward construct(s). This approach allows for simultaneous estimation of multiple regression equations and is ideal for testing theories with multiple observed and/or latent variables (Byrne, 2013). Figure 2 presents the estimated model for each outcome (details in online supplement). SEMs included all available data with bootstrapped 95% CIs (5000 samples). We interpreted a non-significant chi-square value as supporting excellent model fit; evidence of good fit included comparative fit index (CFI) and Tucker-Lewis Index (TLI) ≥ 0.90, root mean square error of approximation (RMSEA) ≤ 0.05 and standardized root mean square residual (SRMR) ≤ 0.08 (Byrne, 2013; Hu & Bentler, 1999).

All indirect effects from weight suppression to eating disorder outcomes were obtained using the “*Model Indirect*” command in Mplus. Specifically, indirect effects pathways were examined from weight suppression, leptin and GLP1 response, via behavioral and self-reported satiation to eating/binge-eating episode size (Model 1); via behavioral and self-reported reward valuation to LOC frequency (Model 2); and all four pathways to global eating disorder severity (Model 3). Indirect effects were interpreted as significant when 95% bootstrapped confidence intervals (CIs) did not cross zero. Sensitivity analyses tested the impact of age, BMI, percent body fat, recruitment before/after pandemic onset, hormonal contraceptive use, SSRI use, current depressive disorder, and current substance use disorder by adding covariate paths to each endogenous variable. Exploratory analyses compared DSM-5 BN-S to controls on variables included in SEM using maximum likelihood estimation for missing values and tested the three SEMs in participants with DSM-5 BN-S.

Power analyses were conducted in R with PowMedR for our least powered analysis (mediation) with multiple imputed data sets. Effect size estimates for *a priori* hypotheses of direct (≥0.22) and indirect pathways (≥0.20) from preliminary studies (Keel et al., 2019) indicated 80% power with *N* = 195.

## Results

**Figure 3.**
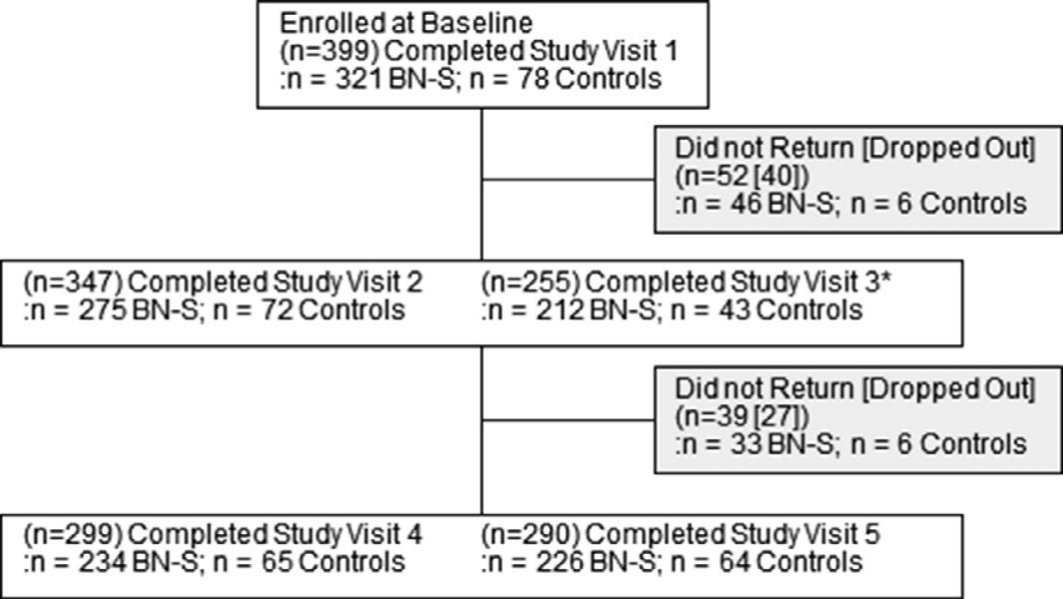
Abbreviation: BN-S = bulimia nervosa and related syndromes. Note: Study Visit 3 was dropped from the protocol for participants enrolled after onset of the COVID-19 pandemic. Participants with Study Visit 3 data include 255 of 301 (85%) enrolled before pandemic onset, including 212 of 256 (84%) with BN-S and 43 of 45 (96%) Control participants. The number who actively dropped out is included in the total number of individuals who did not return. We estimate that *n* = 13 participants were unable to complete visits due to the COVID-19 pandemic.

Participant composition included *n* = 2 (0.5%) American Indian or Alaskan Native, *n* = 14 (3.5%) Asian, *n* = 47 (12%) Black, *n* = 110 (27.6%) Hispanic, *n* = 2 (0.5%) Native Hawaiian or Other Pacific Islander, *n* = 23 (5.8%) Multiracial, and *n* = 311 (77.9%) White (*n* = 221; 55.4% non-Hispanic) (Table 1); 290 of 399 participants (72.7%) completed the full multi-visit protocol (Figure 3).

All results are presented using standardized values in Mplus (STDYX Standardization) (Muthén & Muthén, 2017). Table 2 presents SEM fit indices for each model and path estimates with 95% CIs for all hypothesized pathways. Model 1, predicting eating/binge-eating episode size, provided excellent fit to the data across indices. As hypothesized, greater weight suppression was associated with lower leptin (estimate [SE] = −0.22[0.04]; CIs: −0.31, −0.14; *p* < .001), and lower leptin was associated with lower GLP-1 response (estimate [SE] = 0.16[0.06]; CIs: 0.05, 0.28; *p* < 0.01). Reduced GLP-1 response was associated with reward satiation measured by greater *ad lib* intake (estimate [SE] = −0.15[0.07]; CIs: −0.28, −0.01; *p* < 0.05) and lower self-reported satiation during the fixed meal (estimate [SE] = 0.12[0.06]; CIs: 0.01, 0.23; *p* < 0.05). Moreover, greater *ad lib* intake was associated with larger eating/binge-eating episode size (estimate [SE] = 0.16[0.07]; CI: 0.02, 0.28; *p* < 0.05). Finally, tests of indirect effects for Model 1 supported hypotheses that weight suppression is linked to eating/binge-eating episode size via lower leptin, GLP-1 response, and reward satiation (Table 2).

**Table 2.** Fit statistics for final structural equation models (SEMs) and standardized estimates with bootstrapped 95% confidence intervals for paths in SEMs

	Outcome		
	Model 1 Eating/Binge-eating episode size	Model 2 Loss of control frequency	Model 3 Global eating disorder severity
Fit statistics (threshold)			
Chi-square (df) (*p* > 0.01)	**26.96 (19), *p* =** 0**.11**	72.47(31), *p* < 0.001	161.75(69), *p* < 0.001
CFI/TLI (≥ 0.90)	**0.97/0.96**	**0.92**/0.88	0.89/0.85
RMSEA [90% CI] (≤0.05; [0.00, ≤0.08])	**0.03 [0.00, 0.06]**	0.06 **[0.04, 0.08]**	0.06 **[0.05, 0.07]**
SRMR (≤0.08)	**0.04**	**0.05**	**0.07**
Direct paths: standardized estimates [95% CIs]			
WS to Leptin	**−0.22 [−0.31, −0.14]** ^a^	**−0.22 [−0.31, −0.14]** ^a^	**−0.22 [−0.31, −0.14]** ^a^
Leptin to GLP1	**0.16 [0.05, 0.28]** ^a^	**0.16 [0.05, 0.28]** ^a^	**0.16 [0.04, 0.28]** ^a^
GLP1 to satiation (behavior)	**−0.15 [−0.28, −0.01]** ^a^	NA	**−0.14 [−0.27, −0.01]** ^a^
GLP1 to satiation (self-report)	**0.12 [0.01, 0.23]** ^a^	NA	**0.12 [0.01, 0.23]** ^a^
GLP1 to RV-E (behavior)	NA	−0.01 [−0.14, 0.14]	<−0.01 [−0.14, 0.14]
GLP1 to RV (self report)	NA	0.03 [−0.13, 0.20]	0.03 [−0.13, 0.20]
Satiation (behavior) to outcome	**0.16 [0.02, 0.28]** ^a^	NA	**0.13 [0.01, 0.24]** ^a^
Satiation (self-report) to outcome	−0.06 [−0.17, 0.05]	NA	**0.12 [0.02, 0.23]** ^a^
RV-E (behavior) to outcome	NA	−0.02 [−0.20, 0.14]	0.04 [−0.13, 0.20]
RV (self report) to outcome	NA	0.09 [−0.06, 0.26]	<0.01 [−0.15, 0.16]
Indirect path: 95% CIs			
WS to outcome via satiation (behavior)	**>0.000, 0.003**	NA	**>0.000, 0.002**
WS to outcome via satiation (self-report)	**>0.000**, **0.001**	NA	−0.002, >0.000
WS to outcome via RV-E (behavior)	NA	**>0.000, 0.001**	−0.001, 0.001
WS to outcome via RV (self-report)	NA	−0.001, 0.001	−0.001, 0.001

Abbreviations: CFI = comparative fit index; CIs = Confidence Intervals; GLP1 = Glucagon-like peptide 1 response (total); RMSEA = root mean square error of approximation; RV = reward valuation; RV-E = Reward valuation-effort; SRMR = standardized root mean square residual; TLI = Tucker-Lewis Index; WS = weight suppression.

a *p* ≤ 0.05. Notes: Bold font denotes values that achieve threshold for good fit or statistically significant direct pathways and indirect pathways that do not cross zero.

Models for LOC frequency and global eating disorder severity did not provide good fit across indices (Table 2). Initial paths from higher weight suppression to lower leptin and lower leptin to reduced GLP-1 were observed in these models, and indirect pathway CIs excluded 0 supporting some hypothesized effects. However, Models 2 and 3 supported no significant paths from GLP-1 response to reward valuation measured behaviorally or via self-report and no significant paths between reward valuation and LOC frequency (Model 2) or global eating disorder severity (Model 3). Finally, greater self-reported satiation was associated with higher global eating disorder severity (CIs: 0.02, 0.23), limiting the model’s explanatory value.

### Sensitivity analyses

Including BMI and percent body fat produced good fit for LOC frequency and global eating disorder severity on all indices except chi-square (Supplementary Table S2) and improved fit for all three outcomes (Supplementary Table S3). Resulting direct and indirect pathway estimates support conclusions from main analyses (Supplementary Table S4). Covarying for current mood disorder improved fit for global eating disorder severity (Supplementary Table S3) but did not provide good fit (Supplementary Table S2). No other covariates improved models’ fit (see Tables S2 and S3 in the online supplement for details).

### Exploratory analyses

Compared to controls, BN-S (Supplementary Table S5) differed in expected directions on weight suppression, leptin, *ad lib* intake, reward valuation-effort for food and for game in a fasted state, eating/binge-eating episode size, and global EDE score. Exploratory SEMs in participants with BN-S (*n* = 321; Supplementary Table S6) supported excellent fit for binge-eating episode size and LOC frequency across all indices but poor fit for global eating disorder severity. Path estimates did not change meaningfully from estimates in the full sample, supporting associations with severity of binge-related outcomes in BN-S (Supplementary Table S6).

## Discussion

Findings supported one part of a biobehavioral model for binge-eating severity in BN-S; reward satiation explained associations between weight suppression and severity of eating/binge-eating episode size, via reduced leptin and GLP-1 response. Individuals reporting greater weight loss had lower leptin, lower postprandial GLP-1 response, consumed more food to achieve satiation, reported lower satiation when consuming a fixed amount of food and endorsed consuming larger amounts of food in a limited period of time, with BN-S participants exceeding what most people consume in comparable circumstances. Conversely, the biobehavioral model did not demonstrate good fit for LOC frequency or global eating disorder severity. Furthermore, no significant direct effects emerged for reward valuation.

Significant pathways via reward satiation support both the biobehavioral model and translational approaches, with high conservation from preclinical models to clinical features in humans. Associations between elevated weight suppression, lower leptin, and reduced GLP-1 response may explain why individuals with no apparent energy deficit, based on current body weight, experience reduced ability to achieve satiation, increasing risk for weight gain (Lowe et al., 2018) and BN-S onset (Keel & Heatherton, 2010; Stice, Rohde, Shaw, & Desjardins, 2020) and maintenance (Butryn et al., 2006; Keel & Heatherton, 2010; Lowe et al., 2011).

Experimental GLP-1 manipulation reduces responding on progressive ratio tasks in animals (Balantekin et al., 2024; Woodward et al., 2022). However, current findings did not support associations between postprandial GLP-1 response and progressive ratio task performance or self-reported reward valuation. Partially mirroring current findings, the GLP-1 agonist liraglutide caused no significant changes in self-reported hedonic responses to food despite causing significant weight loss, significant increases in self-reported fullness, and decreases in self-reported hunger in a randomized controlled trial for obesity (Tronieri et al., 2020).

Inadequate model fit for global eating disorder severity likely reflects the range of features measured by the EDE total score, including its emphasis on weight and shape concerns (Thomas, Roberto, & Berg, 2014). Our biobehavioral model aimed to identify biological consequences of weight suppression as novel treatment targets, not to discount the importance of cognitive and affective features. Prior work supported drive for thinness as a significant temporal mediator from greater weight suppression to maintenance of higher bulimia scores (Bodell, Brown, & Keel, 2017), and efforts to advance pharmacological interventions do not replace cognitive behavior therapy as a first-line treatment for BN (Hagan & Walsh, 2021).

Given the cross-sectional design, BMI’s impact on fit indices across models likely reflects its significant positive associations with several variables. Evidence that greater weight suppression is associated with lower versus higher leptin, even controlling for BMI, provides critical support for our model, as does the novel finding linking lower leptin to lower postprandial GLP-1 response in humans. Future research should explore the independent and combined effects of weight suppression and BMI across outcomes given prior associations with body image disturbance (Lavender et al., 2015) and weight trajectory in eating disorders (Piers, Espel‐Huynh, & Lowe, 2019).

Findings have implications for assessment and future clinical trials of GLP-1 agonists for binge eating. Standard eating disorder assessments rarely measure highest adult weight (Schaefer, Crosby, & Machado, 2021). This information required less than 1 min to obtain and contributed to significant findings with biological, behavioral, and clinical data. Results also support testing weight suppression as a moderator of GLP-1 agonist efficacy. A handful of studies have shown GLP-1 agonists reduce Binge Eating Scale scores in patients with obesity (Richards et al., 2023; Robert et al., 2015) or Type 2 diabetes (Da Porto et al., 2020). However, the only double-blind, placebo-controlled randomized trial of liraglutide for BED found no significant effects for binge-eating frequency or remission in 27 patients, despite significantly greater weight loss with liraglutide compared to placebo (Allison et al., 2023). Differences in weight suppression at intake as well as differences that may emerge during treatment could potentially obscure the impact of GLP-1 agonist treatment on binge eating.

Study strengths include the large, ethnically and racially diverse sample, and good retention across visits. High fidelity in translating behavioral assays from preclinical studies, evidence of convergent and discriminant validity of measures and their strong psychometric properties are additional strengths. Analyses reduced impact of attrition through bootstrapping and imputation, and sensitivity analyses supported minimal impact of potential confounds, such as comorbid disorders, SSRI or hormonal contraceptive use, further improving generalizability. These strengths ensured a rigorous test of our biobehavioral model’s *a priori* hypotheses, produced excellent model fit for significant pathways from weight suppression to eating/binge-eating episode size via leptin, GLP-1 function, and reward satiation. Exploratory analyses supported associations with binge severity in the BN-S group, despite the smaller sample size and restricted variable range in this subsample compared to the full sample.

Limitations to generalizability include restricting eligibility to women who were free of medical morbidity and were medication free for biobehavioral assessments. These criteria were necessary to reduce confounds but likely reduced inclusion of those with ANbp and BED because medical morbidity is more common in both (Udo & Grilo, 2019). Despite this limitation to generalizability, the current sample may better represent those for whom future GLP-1 agonist treatment would be warranted by its efficacy for binge eating, specifically in the absence of its indications for medical conditions or obesity or its clear contraindication for patients who are medically underweight. Moreover, future work on GLP-1 agonists in eating disorders should address potential iatrogenic effects because misuse of medication for weight loss is a symptom of BN and related syndromes.

Nonsignificant reward valuation findings may reflect design limitations. Reward valuation was not consistently measured the same day as postprandial GLP-1 response (Figure 1). Assessment timing or protocol changes may have reduced associations with this construct. Alternatively, another reward valuation-effort measure (Treadway et al., 2009) may have revealed stronger associations with GLP-1 response and LOC frequency. Relatedly, alternative LOC eating measures (Bodell et al., 2018), focused on craving strength or degree of LOC, may better capture GLP-1 effects (Badulescu et al., 2024).

Importantly, current findings focused on physiological GLP-1 function versus pharmacological GLP-1 manipulations. Although supra-physiological GLP-1 levels may produce large behavioral effects, regardless of underlying pathophysiology, precision medicine requires identifying ‘what works in whom’ (Insel, 2014). In addition to weight suppression’s potential moderating effect, results suggest blunted GLP-1 function may associate most with disorders featuring excessive food intake. Although weight suppression predicted restricting AN and purging disorder onset (Stice et al., 2020), and lower leptin was observed in both (Germain et al., 2007; Jimerson, Wolfe, Carroll, & Keel, 2010), neither has demonstrated blunted postprandial GLP-1 responses (Dossat et al., 2015; Germain et al., 2007). Current findings support need for a separate biobehavioral model for restricting AN and purging disorder. Given our small effect sizes, future studies should employ a developmental measure of weight suppression (Lowe, Singh, Rosenbaum, & Mayer, 2024) and examine other potential mechanisms, including ghrelin, insulin, and glucose-dependent insulinotropic polypeptide to develop combination treatments, which may achieve therapeutic benefits at lower doses with fewer side effects (Woodward et al., 2022). Finally, our cross-sectional tests of concurrent associations do not permit temporal or causal inferences. A prospective design should examine our biobehavioral model’s predictions for BN-S maintenance (Keel et al., 2019).

## Conclusions

This study supported links from weight suppression to eating/binge-eating episode size via reduced leptin, GLP-1 response, and reward satiation. The specific link between GLP-1 response and excessive food intake supports DSM-5 over ICD-11 criteria for binge eating because only DSM-5 criteria require excessive food intake. Furthermore, excellent model fit in a sample comprising DSM-5 BN and OSFED BN-low frequency/duration supports potential adjustments to frequency and duration criteria in future diagnostic criteria. Conclusions support how the RDoC framework complements existing categorical diagnostic systems instead of replacing them.

## Supporting information

Keel et al. supplementary material Keel et al. supplementary material

## Supplementary material

The supplementary material for this article can be found at http://doi.org/10.1017/S0033291725100871.

## References

[r1] Allison, K. C., Chao, A. M., Bruzas, M. B., McCuen-Wurst, C., Jones, E., McAllister, C., … Tronieri, J. S. (2023). A pilot randomized controlled trial of liraglutide 3.0 mg for binge eating disorder. Obesity Science and Practice, 9(2), 127–136. 10.1002/osp4.619. 37034559 PMC10073825

[r2] Anini, Y., & Brubaker, P. L. (2003). Role of leptin in the regulation of glucagon-like peptide-1 secretion. Diabetes, 52(2), 252–259. http://www.ncbi.nlm.nih.gov/entrez/query.fcgi?cmd=Retrieve&db=PubMed&dopt=Citation&list_uids=12540594 12540594 10.2337/diabetes.52.2.252

[r3] Badulescu, S., Tabassum, A., Le, G. H., Wong, S., Phan, L., Gill, H., & Mansur, R. (2024). Glucagon-like peptide 1 agonist and effects on reward behaviour: A systematic review. Physiology & Behavior, 283, 114622. 10.1016/j.physbeh.2024.114622 38945189

[r4] Balantekin, K. N., Kretz, M. J., & Mietlicki-Baase, E. G. (2024). The emerging role of glucagon-like peptide 1 in binge eating. The Journal of Endocrinology, 262(1), e230405. 10.1530/JOE-23-0405. 38642585 PMC11156433

[r5] Bodell, L. P., Brown, T. A., & Keel, P. K. (2017). Weight suppression predicts bulimic symptoms at 20-year follow-up: The mediating role of drive for thinness. Journal of Abnormal Psychology, 126(1), 32. 27808544 10.1037/abn0000217 PMC5215971

[r6] Bodell, L. P., Forney, K. J., Chavarria, J., Keel, P. K., & Wildes, J. E. (2018). Self‐report measures of loss of control over eating: Psychometric properties in clinical and non‐clinical samples. International Journal of Eating Disorders, 51(11), 1252–1260. 30265751 10.1002/eat.22957 PMC11037076

[r7] Bodell, L. P., & Keel, P. K. (2015). Weight suppression in bulimia nervosa: Associations with biology and behavior. Journal of Abnormal Psychology, 124(4), 994–1002. 10.1037/abn0000077. 26191637 PMC4658277

[r8] Boneva-Asiova, Z., & Boyanov, M. A. (2008). Body composition analysis by leg-to-leg bioelectrical impedance and dual-energy X-ray absorptiometry in non-obese and obese individuals. Diabetes, Obesity & Metabolism, 10(11), 1012–1018. 10.1111/j.1463-1326.2008.00851.x. 18435776

[r9] Bulik, C. M., & Brinded, E. C. (1994). The effect of food deprivation on the reinforcing value of food and smoking in bulimic and control women. Physiology & Behavior, 55(4), 665–672. 10.1016/0031-9384(94)90042-6 8190792

[r10] Butryn, M. L., Juarascio, A., & Lowe, M. R. (2011). The relation of weight suppression and BMI to bulimic symptoms. The International Journal of Eating Disorders, 44(7), 612–617. 10.1002/eat.20881. 21997424 PMC4852137

[r11] Butryn, M. L., Lowe, M. R., Safer, D. L., & Agras, W. S. (2006). Weight suppression is a robust predictor of outcome in the cognitive-behavioral treatment of bulimia nervosa. Journal of Abnormal Psychology, 115(1), 62–67. 10.1037/0021-843x.115.1.62. 16492096

[r12] Byrne, B. M. (2013). Structural equation modeling with Mplus: Basic concepts, applications, and programming. Routledge.

[r13] Cassioli, E., Lucherini Angeletti, L., Rossi, E., Selvi, G., Riccardi, E., Siviglia, S., … Castellini, G. (2024). Leptin levels in acute and recovered eating disorders: An arm-based network meta-analysis. European Eating Disorders Review. 10.1002/erv.3163 PMC11965547 39643920

[r14] Crow, S. J., Peterson, C. B., Swanson, S. A., Raymond, N. C., Specker, S., Eckert, E. D., & Mitchell, J. E. (2009). Increased mortality in bulimia nervosa and other eating disorders. American Journal of Psychiatry, 166(12), 1342–1346. 19833789 10.1176/appi.ajp.2009.09020247

[r15] Cuthbert, B. N., & Insel, T. R. (2013). Toward the future of psychiatric diagnosis: The seven pillars of RDoC. BMC Medicine, 11, 1–8. 23672542 10.1186/1741-7015-11-126 PMC3653747

[r16] Da Porto, A., Casarsa, V., Colussi, G., Catena, C., Cavarape, A., & Sechi, L. (2020). Dulaglutide reduces binge episodes in type 2 diabetic patients with binge eating disorder: A pilot study. Diabetes and Metabolic Syndrome: Clinical Research and Reviews, 14(4), 289–292. 10.1016/j.dsx.2020.03.009. 32289741

[r17] Dossat, A. M., Bodell, L. P., Williams, D. L., Eckel, L. A., & Keel, P. K. (2015). Preliminary examination of glucagon-like peptide-1 levels in women with purging disorder and bulimia nervosa. The International Journal of Eating Disorders, 48(2), 199–205. 10.1002/eat.22264. 24590464 PMC4155021

[r18] Epstein, L. H., Leddy, J. J., Temple, J. L., & Faith, M. S. (2007). Food reinforcement and eating: A multilevel analysis. Psychological Bulletin, 133(5), 884–906. 10.1037/0033-2909.133.5.884. 17723034 PMC2219695

[r19] Fairbum, C. G., Cooper, Z., & O’Connor, M. (2014). Eating disorders examination 17.0D. Available from: https://www.cbte.co/download/ede-17-0d/

[r20] First, M., Williams, J., Karg, R., & Spitzer, R. (2014). Structured clinical interview for DSM-5 disorders–research version (SCID-5-RV). American Psychiatric Assocation.

[r21] Fornaro, M., Mondin, A. M., Billeci, M., Fusco, A., De Prisco, M., Caiazza, C., & de Bartolomeis, A. (2023). Psychopharmacology of eating disorders: Systematic review and meta-analysis of randomized controlled trials. Journal of Affective Disorders, 338, 526–545. 37393954 10.1016/j.jad.2023.06.068

[r22] Geliebter, A., Melton, P. M., McCray, R. S., Gallagher, D. R., Gage, D., & Hashim, S. A. (1992). Gastric capacity, gastric emptying, and test-meal intake in normal and bulimic women. The American Journal of Clinical Nutrition, 56(4), 656–661. http://www.ncbi.nlm.nih.gov/entrez/query.fcgi?cmd=Retrieve&db=PubMed&dopt=Citation&list_uids=1414964 1414964 10.1093/ajcn/56.4.656

[r23] Germain, N., Galusca, B., Le Roux, C. W., Bossu, C., Ghatei, M. A., Lang, F., & Estour, B. (2007). Constitutional thinness and lean anorexia nervosa display opposite concentrations of peptide YY, glucagon-like peptide 1, ghrelin, and leptin. The American Journal of Clinical Nutrition, 85(4), 967–971. 17413094 10.1093/ajcn/85.4.967

[r24] Hadigan, C. M., Walsh, B. T., Devlin, M. J., LaChaussee, J. L., & Kissileff, H. R. (1992). Behavioral assessment of satiety in bulimia nervosa. Appetite, 18(3), 233–241. http://www.ncbi.nlm.nih.gov/entrez/query.fcgi?cmd=Retrieve&db=PubMed&dopt=Citation&list_uids=1510465 1510465 10.1016/0195-6663(92)90200-p

[r25] Hagan, K. E., & Walsh, B. T. (2021). State of the art: The therapeutic approaches to bulimia nervosa. Clinical Therapeutics, 43(1), 40–49. 33358256 10.1016/j.clinthera.2020.10.012 PMC7902447

[r26] Hahs-Vaughn, D. L., & Lomax, R. (2020). An introduction to statistical concepts. Routledge.

[r27] Hu, L.-t., & Bentler, P. M. (1999). Cutoff criteria for fit indexes in covariance structure analysis: Conventional criteria versus new alternatives. Structural Equation Modeling: A Multidisciplinary Journal, 6(1), 1–55.

[r28] Insel, T. R. (2014). The NIMH research domain criteria (RDoC) project: Precision medicine for psychiatry. American Journal of Psychiatry, 171(4), 395–397. 24687194 10.1176/appi.ajp.2014.14020138

[r29] Jimerson, D. C., Wolfe, B. E., Carroll, D. P., & Keel, P. K. (2010). Psychobiology of purging disorder: Reduction in circulating leptin levels in purging disorder in comparison with controls. International Journal of Eating Disorders, 43(7), 584–588. 19722179 10.1002/eat.20738 PMC2891937

[r30] Keel, P. K., Bodell, L. P., Ali, S. I., Starkey, A., Trotta, J., Luxama, J. W., & Williams, D. L. (2025). Examining weight suppression, leptin levels, glucagon-like peptide 1 response, and reward-related constructs in severity and maintenance of bulimic syndromes: Protocol and sample characteristics for a cross-sectional and longitudinal study. JMIR Research Protocols, 14, e66554. 10.2196/66554. 40198107 PMC12015349

[r31] Keel, P. K., Bodell, L. P., Forney, K. J., Appelbaum, J., & Williams, D. (2019). Examining weight suppression as a transdiagnostic factor influencing illness trajectory in bulimic eating disorders. Physiology & Behavior, 208, 112565. 31153878 10.1016/j.physbeh.2019.112565 PMC6636832

[r32] Keel, P. K., Bodell, L. P., Haedt‐Matt, A. A., Williams, D. L., & Appelbaum, J. (2017). Weight suppression and bulimic syndrome maintenance: Preliminary findings for the mediating role of leptin. International Journal of Eating Disorders, 50(12), 1432–1436. 29044587 10.1002/eat.22788 PMC5752142

[r33] Keel, P. K., Eckel, L. A., Hildebrandt, B. A., Haedt-Matt, A. A., Appelbaum, J., & Jimerson, D. C. (2018). Disturbance of gut satiety peptide in purging disorder. The International Journal of Eating Disorders, 51(1), 53–61. 10.1002/eat.22806. 29219202 PMC13222533

[r34] Keel, P. K., Haedt, A., & Edler, C. (2005). Purging disorder: An ominous variant of bulimia nervosa? The International Journal of Eating Disorders, 38(3), 191–199. 10.1002/eat.20179. 16211629

[r35] Keel, P. K., Haedt-Matt, A. A., Hildebrandt, B., Bodell, L. P., Wolfe, B. E., & Jimerson, D. C. (2018). Satiation deficits and binge eating: Probing differences between bulimia nervosa and purging disorder using an ad lib test meal. Appetite, 127, 119–125. 10.1016/j.appet.2018.04.009. 29654850 PMC5994372

[r36] Keel, P. K., & Heatherton, T. F. (2010). Weight suppression predicts maintenance and onset of bulimic syndromes at 10-year follow-up. Journal of Abnormal Psychology, 119(2), 268–275. 10.1037/A0019190. 20455599 PMC2869470

[r37] Keel, P. K., Kennedy, G. A., Rogers, M. L., Joyner, K. J., Bodell, L. P., Forney, K. J., & Duffy, M. E. (2022). Reliability and validity of a transdiagnostic measure of reward valuation effort. Psychological Assessment, 34(5), 419. 35025580 10.1037/pas0001107 PMC10026017

[r38] Keel, P. K., Wolfe, B. E., Liddle, R. A., De Young, K. P., & Jimerson, D. C. (2007). Clinical features and physiological response to a test meal in purging disorder and bulimia nervosa. Archives of General Psychiatry, 64(9), 1058–1066. 10.1001/archpsyc.64.9.1058. 17768271

[r39] Lavender, J. M., Shaw, J. A., Crosby, R. D., Feig, E. H., Mitchell, J. E., Crow, S. J., & Lowe, M. R. (2015). Associations between weight suppression and dimensions of eating disorder psychopathology in a multisite sample. Journal of Psychiatric Research, 69, 87–93. 26343599 10.1016/j.jpsychires.2015.07.021 PMC4561862

[r40] Lowe, M. R. (1993). The effects of dieting on eating behavior: A three-factor model. Psychological Bulletin, 114(1), 100–121. 10.1037/0033-2909.114.1.100. 8346324

[r41] Lowe, M. R., Berner, L. A., Swanson, S. A., Clark, V. L., Eddy, K. T., Franko, D. L., & Herzog, D. B. (2011). Weight suppression predicts time to remission from bulimia nervosa. Journal of Consulting and Clinical Psychology, 79(6), 772–776. 10.1037/a0025714. 22004302

[r42] Lowe, M. R., Piers, A. D., & Benson, L. (2018). Weight suppression in eating disorders: A research and conceptual update. Current Psychiatry Reports, 20, 1–12. 30155651 10.1007/s11920-018-0955-2

[r43] Lowe, M. R., Singh, S., Rosenbaum, M., & Mayer, L. (2024). Physiological, body composition, and body mass measures show that a developmental measure of weight suppression is more valid than the traditional measure. International Journal of Eating Disorders, 57(7), 1599–1608. 38597163 10.1002/eat.24210 PMC11949195

[r44] Lowe, M. R., Thomas, J. G., Safer, D. L., & Butryn, M. L. (2007). The relationship of weight suppression and dietary restraint to binge eating in bulimia nervosa. The International Journal of Eating Disorders, 40(7), 640–644. 10.1002/eat.20405. 17607698

[r45] Mitchell, J. E., Crow, S., Peterson, C. B., Wonderlich, S., & Crosby, R. D. (1998). Feeding laboratory studies in patients with eating disorders: A review. The International Journal of Eating Disorders, 24(2), 115–124. 10.1002/(sici)1098-108x(199809)24:2<115::aid-eat1>3.0.co;2-h. 9697010

[r46] Muthén, L. K., & Muthén, B. O. (2017). Mplus user’s guide (8th ed.) Muthén & Muthén.

[r47] Piers, A. D., Espel‐Huynh, H. M., & Lowe, M. R. (2019). The independent and interacting effects of weight suppression and admission body mass index on treatment weight change in patients with anorexia nervosa or bulimia nervosa. International Journal of Eating Disorders, 52(11), 1301–1309. 31392766 10.1002/eat.23149

[r48] Richards, J., Bang, N., Ratliff, E. L., Paszkowiak, M. A., Khorgami, Z., Khalsa, S. S., & Simmons, W. K. (2023). Successful treatment of binge eating disorder with the GLP-1 agonist semaglutide: A retrospective cohort study. Obesity Pillars, 7, 100080. 37990682 10.1016/j.obpill.2023.100080 PMC10661993

[r49] Robert, S. A., Rohana, A. G., Shah, S. A., Chinna, K., Wan Mohamud, W. N., & Kamaruddin, N. A. (2015). Improvement in binge eating in non-diabetic obese individuals after 3 months of treatment with liraglutide – a pilot study. Obesity Research & Clinical Practice, 9(3), 301–304. 10.1016/j.orcp.2015.03.005. 25870084

[r50] Schaefer, L. M., Crosby, R. D., & Machado, P. P. (2021). A systematic review of instruments for the assessment of eating disorders among adults. Current Opinion in Psychiatry, 34(6), 543–562. 34475351 10.1097/YCO.0000000000000746 PMC8645259

[r51] Schaumberg, K., Anderson, L. M., Reilly, E. E., Gorrell, S., Anderson, D. A., & Earleywine, M. (2016). Considering alternative calculations of weight suppression. Eating Behaviors, 20, 57–63. 26643591 10.1016/j.eatbeh.2015.11.003

[r52] Schebendach, J., Broft, A., Foltin, R. W., & Walsh, B. T. (2013). Can the reinforcing value of food be measured in bulimia nervosa? Appetite, 62, 70–75. 10.1016/j.appet.2012.11.009. 23178173 PMC3552030

[r53] Stefanakis, K., Upadhyay, J., Ramirez-Cisneros, A., Patel, N., Sahai, A., & Mantzoros, C. S. (2024). Leptin physiology and pathophysiology in energy homeostasis, immune function, neuroendocrine regulation and bone health. Metabolism – Clinical and Experimental, 161. 10.1016/j.metabol.2024.156056. 39481533

[r54] Stice, E., Rohde, P., Shaw, H., & Desjardins, C. (2020). Weight suppression increases odds for future onset of anorexia nervosa, bulimia nervosa, and purging disorder, but not binge eating disorder. The American Journal of Clinical Nutrition, 112(4), 941–947. 32534455 10.1093/ajcn/nqaa146 PMC7528557

[r55] Sun, G., French, C. R., Martin, G. R., Younghusband, B., Green, R. C., Xie, Y. G., & Zhang, H. (2005). Comparison of multifrequency bioelectrical impedance analysis with dual-energy X-ray absorptiometry for assessment of percentage body fat in a large, healthy population. The American Journal of Clinical Nutrition, 81(1), 74–78. 10.1093/ajcn/81.1.74. 15640463

[r56] Thomas, J. J., Roberto, C. A., & Berg, K. C. (2014). The eating disorder examination: A semi-structured interview for the assessment of the specific psychopathology of eating disorders. Advances in Eating Disorders: Theory, Research and Practice, 2(2), 190–203.

[r57] Treadway, M. T., Buckholtz, J. W., Schwartzman, A. N., Lambert, W. E., & Zald, D. H. (2009). Worth the ‘EEfRT’? The effort expenditure for rewards task as an objective measure of motivation and anhedonia. PLoS One, 4(8), e6598. 19672310 10.1371/journal.pone.0006598 PMC2720457

[r58] Tronieri, J. S., Wadden, T. A., Walsh, O., Berkowitz, R. I., Alamuddin, N., Gruber, K., & Chao, A. M. (2020). Effects of liraglutide on appetite, food preoccupation, and food liking: Results of a randomized controlled trial. International Journal of Obesity, 44(2), 353–361. 30926955 10.1038/s41366-019-0348-6 PMC6766432

[r59] Udo, T., & Grilo, C. M. (2019). Psychiatric and medical correlates of DSM-5 eating disorders in a nationally representative sample of adults in the United States. The International Journal of Eating Disorders, 52(1), 42–50. 10.1002/eat.23004. 30756422

[r60] Udo, T., & Grilo, C. M. (2022). Epidemiology of eating disorders among US adults. Current Opinion in Psychiatry, 35(6), 372–378. 35855509 10.1097/YCO.0000000000000814

[r61] Williams, K. W., & Elmquist, J. K. (2012). From neuroanatomy to behavior: Central integration of peripheral signals regulating feeding behavior. Nature Neuroscience, 15(10), 1350–1355. 10.1038/Nn.3217. 23007190 PMC3763714

[r62] Wolfe, B., Metzger, E., & Jimerson, D. (2002). Serotonin and satiety in bulimia nervosa. Biological Psychiatry, 51(8), 12s–12s.

[r63] Woodward, O. R. M., Gribble, F. M., Reimann, F., & Lewis, J. E. (2022). Gut peptide regulation of food intake – evidence for the modulation of hedonic feeding. The Journal of Physiology, 600(5), 1053–1078. 10.1113/JP280581. 34152020

