# Supplementary material for: Test of a biobehavioral model linking weight suppression to binge-eating severity via leptin and glucagon-like peptide 1 in bulimia nervosa and related syndromes in women
Source: Psychol Med. 2025 Jun 27;55:e177. doi: 10.1017/S0033291725100871 (PMC12234017; doi:10.1017/S0033291725100871)
Supplement: Keel et al. supplementary material [file S0033291725100871sup001.docx]

Supplement for Keel et al., Test of a Biobehavioral Model Linking Weight Suppression to Binge-Eating Severity via Leptin and Glucagon-Like Peptide 1 in Bulimia Nervosa and Related Syndromes in Women

**Correlation Analyses**

Correlation analyses were conducted in SPSS version 29. Correlations were conducted using bootstrap (1000 samples). Bivariate Pearson correlations were run between continuous variables. Non-parametric correlations using Spearman’s rho were conducted with RV-E variables to account for RV-E as an ordinal variable.

Correlations among study variables are included in Table S1. As predicted, higher weight suppression was correlated with lower fasting leptin (r= -0.23; p<.001; 95% CI: -0.31, -0.14), which was associated with lower GLP-1 response (r= 0.16; p<.01; 95% CI: 0.05, 0.29). Reduced GLP-1 response was correlated with greater *ad lib* intake (r= -0.15; p=.02; 95% CI: -0.28, -0.01) and lower self-reported fullness (r= 0.12; p<.05; 95% CIs: 0.01, 0.23). Greater *ad lib* intake was correlated with larger eating/binge-eating episode size (r= 0.16; p<.01; 95% CI: 0.03, 0.29). Contrary to hypotheses, there were no significant correlations between GLP-1 response and reward valuation-effort (Spearman’s rho=-0.02-.02; ps> .05) or reward valuation (self-report) (rs=-0.08-0.10; ps>.05). Moreover, there were no significant associations between reward valuation measures and LOC frequency (ps>.05) (Table S1).

**SEM Analyses**

Variable Transformations and Latent Variables. To control for interassay variability, standardized residuals for leptin and GLP-1 values were saved from models controlling for assay in which samples were run. Residuals were used as observed variables in SEMs. To achieve model convergence, PR task breakpoints and VAS AUC ratings for satiation were rescaled by dividing all individual values by 100 and 1000, respectively, and VAS wanting ratings were rescaled by dividing all individual values by 10.

Latent variables were created for reward valuation (self-report; RV), reward valuation-effort (RV-E), and reward satiation (self-report). Specifically, breakpoints on each of three PR tasks were used as indicators for RV-E. The three VAS self-reported ‘wanting’ ratings administered just prior to each PR task were used as indicators for RV (self-report). Finally, the AUC VAS ratings for fullness, satiation, and hunger (reversed scored) were used as the three indicators for reward satiation (self-report). The error variance of the RV-E and RV (self-report) latent factors were fixed to one.

Initial Model Convergence and Modification Indices. The initial model tested for eating/binge-eating episode size (Model 1) did not converge due to negative residual variance for self-reported fullness. After setting this parameter to zero, model fit was excellent (see Table S2) and no modification indices were suggested. Mplus output for this model indicated that the analysis may have multiple solutions. This issue was explored using random starts (20). Of 20 random start values, 17 runs converged, and the best fit function was replicated with one random starts number unperturbed.

Initial model fit for LOC frequency (Model 2) was inadequate (Chi-square(df)=110.39, *p*<.001; CFI/TLI= 0.84/0.78; RMSEA=0.08; SRMR=0.06). Modification indices suggested allowing behavioral and self-report assessments of food reward valuation (i.e., M&M PR task breakpoint and VAS wanting rating for M&Ms) to correlate to improve model fit (Chi-square(df)=72.47(31), *p*<.001; CFI/TLI= 0.92/0.88; RMSEA=0.06; SRMR=0.05). This modification is supported by theory underlying multi-unit measurement of reward valuation. Standardized estimates and 95% confidence intervals for paths in this model are presented in Table 2. Modification indices also suggested adding a direct path from weight suppression to LOC frequency, which improved model fit (Chi-square(df)=61.65(30), *p*<.001; CFI/TLI= 0.94/0.91; RMSEA=0.05; SRMR=0.05). Importantly, this model does not provide a direct test of our *a priori* hypotheses. Instead, it suggests that weight suppression may be linked to LOC frequency via variables not included in our model. Finally, as with Model 1, Mplus output for Model 2 indicated that the analysis may have multiple solutions. This was explored using random starts (20), and the best fit function was replicated with one random starts number unperturbed.

Based on modifications to Models 1 and 2, the initial test of Model 3 included a correlated path between behavioral and self-report assessments of food reward valuation and residual variance of self-reported fullness VAS rating set to zero. Initial model fit was inadequate (Chi-square(df)=179.12(70), *p*<.001; CFI/TLI= 0.87/0.82; RMSEA=0.06; SRMR=0.08). Modification indices suggested adding a correlated path between satiation (behavioral; ad lib test meal intake) and reward valuation (behavioral) latent variable to improve model fit. This modification is consistent with hypothesized influence of GLP-1 on both outcomes. Table 2 presents fit statistics and standardized estimates of paths with these modifications. Similar to Model 2, modification indices for Model 3 suggested adding a direct path from weight suppression to global eating disorder severity (EDE score). As with Model 2, this modification does not permit direct tests of our *a priori* hypotheses. In addition, even with this modification, model fit remained inadequate (Chi-square(df)=148.18(68), *p*<.001; CFI/TLI= 0.90/0.87; RMSEA=0.05; SRMR=0.07). Finally, multiple solutions were explored using random starts (20). Of 20 random start values, 17 runs converged, and the best fit function was replicated with one random starts number unperturbed.

Sensitivity Analyses. Sensitivity analyses were conducted to determine the potential impact of eight specific variables on model fit and direct paths for each BN-S outcome (i.e., LOC frequency, eating/binge-eating episode size, global eating disorder severity). Specifically, age, body mass index (BMI), percent body fat, recruitment status (i.e., before or after COVID-19 pandemic), hormonal contraceptive use, SSRI use, mood disorder diagnosis, or substance use disorder diagnosis were included in SEMs as individual covariates in separate models with paths to each endogenous variable (i.e., every variable except weight suppression). Fit statistics for these models are included in Table S2. Inclusion of BMI or percent body fat as covariates improved model fit indices for LOC frequency and global eating disorder severity, such that with these covariates, these models provided good fit to the data across all fit indices except the chi-square statistic, which remained significant (Table S2). We also compared models with and without covariates using Bayesian Information Criterion (BIC) values (Table S3). Change in BIC values (>200) favored models with BMI or body fat as covariates for all outcomes (i.e., LOC frequency, eating/binge-eating episode size, and global eating disorder severity). Change in BIC values (>25) also favored the model with current mood disorder diagnosis as a covariate for global eating disorder severity (Model 3); however, fit statistic thresholds were not met with the exception of SRMR <0.08. Model comparisons for all other covariates favored the original, more parsimonious models. As such, we only considered models with BMI or percent body fat as having superior model fit compared to our original hypothesized models. Notably, results of models with percent body fat as a covariate were identical to those with BMI as a covariate, reflecting the fact that percent body fat and BMI were highly correlated in our sample (*r*=0.90, *p*<.001; 95% CI: 0.88, 0.92). Given evidence that models with BMI represented better fit to the data than models with percent body fat (i.e., change in BIC between models with BMI vs. percent body fat range 31.1-41.0; Table S3), we present models with BMI (see Table S4).

As noted in Table S4, BMI was significantly positively associated with leptin levels, *ad lib* test meal intake, RV-E, LOC frequency, eating/binge-eating episode size, and global eating disorder severity. All the significant direct pathways in the original models (i.e., Models 1, 2, and 3) remained significant when accounting for BMI except the pathways from *ad lib* intake (behavioral satiation) to eating/binge-eating episode size (two-tailed *p*=.10) and *ad lib* intake to eating disorder global severity (*p*=.27). The indirect pathways from weight suppression to eating/binge-eating episode size or eating disorder severity via satiation (*ad lib* intake) remained significant, while the confidence intervals for the indirect pathways from weight suppression to LOC frequency crossed zero. Notably, the indirect pathway from weight suppression to eating/binge-eating episode size via satiation (fullness) and the indirect pathway from weight suppression to eating disorder severity via reward valuation (self-report) emerged as significant. However, caution should be taken when interpreting these findings, given the small effects and non-significant paths between fullness and eating/binge-eating episode size and between reward valuation and eating disorder severity.

**Comparisons of Diagnostic Groups**

Exploratory analyses examined whether diagnostic groups differed in key variables included in our model given that prior studies have taken a categorical, DSM-based approach to probe biobehavioral disruptions in eating disorders. Inclusion criteria were intended to capture a transdiagnostic sample and recruitment did not depend on achieving a minimum number of participants in any specific DSM-5 eating disorder diagnosis. Statistical power to detect medium and small effect sizes varies across comparisons, with adequate power in comparisons of individuals with eating disorders versus control participants (Table S5). Parametric tests (e.g., independent samples t-test or ANOVA) were conducted with continuous variables and non-parametric tests (i.e., Mann-Whitney U or Kruskal-Wallis) were used for to compare diagnostic groups on RV-E due to the ordinal nature of responses.

Consistent with their diagnosis, individuals with BN-S had significantly higher EDE global scores (Cohen’s d= 3.58) and larger eating/binge-eating episode size (Cohen’s d=1.25) compared to control participants. Because controls were required to be free of eating disorder symptoms, including subjective bulimic episodes, purging behaviors, etc., no comparisons were possible on these variables. Consistent with our hypothesized model and prior studies, individuals with BN-S also demonstrated greater weight suppression (Cohen’s d=0.46), greater intake during the *ad lib* meal (Cohen’s d=0.39), and greater RV-E on the food reward task and fasted game task (η*2*= 0.02 and 0.03, respectively) compared to the control participants. Finally, individuals with BN-S had higher BMIs (Cohen’s d= 0.69), greater percent body fat (Cohen’s d= 0.68), and higher leptin levels than control participants (η^2^=0.06).

**Table S1**. Correlations among Study Variables

|  | 1 | 2^a^ | 3^a^ | 4 | 5 | 6 | 7 | 8^b^ | 9^b^ | 10^b^ | 11 | 12 | 13 | 14 | 15 | 16 |
| --- | --- | --- | --- | --- | --- | --- | --- | --- | --- | --- | --- | --- | --- | --- | --- | --- |
| 1. Weight Suppression | -- |  |  |  |  |  |  |  |  |  |  |  |  |  |  |  |
| 2. Leptin^a^ | -.23* | -- |  |  |  |  |  |  |  |  |  |  |  |  |  |  |
| 3. GLP-1^a^ | -.10 | .16* | -- |  |  |  |  |  |  |  |  |  |  |  |  |  |
| 4. Ad Lib Intake | .06 | .11 | -.15* | -- |  |  |  |  |  |  |  |  |  |  |  |  |
| 5. VAS hunger (reversed) | -.04 | .08 | .07 | -.16* | -- |  |  |  |  |  |  |  |  |  |  |  |
| 6. VAS fullness | -.06 | .04 | .12* | -.16* | .60* | -- |  |  |  |  |  |  |  |  |  |  |
| 7. VAS satiation | -.01 | .03 | -.01 | -.01 | .30* | .51* | -- |  |  |  |  |  |  |  |  |  |
| 8. RV-E food reward^b^ | .01 | .20* | .02 | .40* | -.23* | -.19* | -.03 | -- |  |  |  |  |  |  |  |  |
| 9. RV-E game reward fed state^b^ | -.01 | .03 | -.02 | .21* | -.12* | -.07 | .05 | .40* | -- |  |  |  |  |  |  |  |
| 10. RV-E game reward fasted state^b^ | .07 | .07 | <.01 | .26* | -.10 | -.12* | .03 | .49* | .60* | -- |  |  |  |  |  |  |
| 11. VAS want food reward | .05 | .05 | -.08 | .21* | -.19* | -.08 | .02 | .45* | .23* | .15* | -- |  |  |  |  |  |
| 12. VAS want game reward fed | .05 | -.04 | -.04 | .05 | -.19* | -.04 | .14* | .20* | .35* | .20* | .27* | -- |  |  |  |  |
| 13. VAS want game reward fasted | <.01 | .09 | .10 | .03 | -.09 | .01 | .06 | .23* | .30* | .28* | .31* | .51* | -- |  |  |  |
| 14. Eating/binge-eating episode size | .12* | .10 | <.01 | .16* | -.03 | -.09 | -.02 | .07 | .09 | .05 | -.01 | .04 | .06 | -- |  |  |
| 15. LOC frequency | .17* | .13* | -.07 | .05 | .13* | .09 | .01 | .05 | -.03 | .07 | -.01 | .03 | .08 | .42* | -- |  |
| 16. EDE | .18* | .23* | .03 | .12* | .14* | .10 | .09 | .01 | .01 | .08 | -.01 | .03 | .02 | .50* | .69* | -- |

Abbreviations: EDE= Eating Disorder Examination; GLP-1=Glucagon-like peptide 1; LOC frequency= loss of control frequency; RV-E= reward valuation effort; VAS= visual analog scale

Notes: *p<.05; a= used standardized residuals for leptin and GLP-1 values that controlled for assay in which plasma samples were run; b=non-parametric correlations using Spearman’s rho to account for RV-E as ordinal variable

**Table S2.** Sensitivity Analyses: Fit Statistics for Structural Equation Models with Covariates

|  | **Outcome** | | |
| --- | --- | --- | --- |
| **Covariates** | **Model 1**  **Eating/Binge Eating**  **Episode Size** | **Model 2**  **Loss of Control Frequency** | **Model 3**  **Global Eating Disorder Severity (EDE)** |
| **1. Age** | | | |
| Chi-square (df) | **30.94(21), p=.07** | 77.83(35), p<.001 | 172.03(75), p<.001 |
| CFI/TLI | **.96/.94** | **.92**/.87 | .88/.83 |
| RMSEA (90% CI) | **.03 (.00-.06)** | .06 **(.04-.07)** | .06 **(.05-.07)** |
| SRMR | **.04** | **.05** | **.07** |
| **2. BMI** | | | |
| Chi-square (df) | **22.76(21), p=.36** | 64.96(35), p<.001 | 144.74(75), p<.001 |
| CFI/TLI | **.99/.99** | **.96/.94** | **.94/.91** |
| RMSEA (90% CI) | **.01 (.00-.05)** | **.05 (.03-.06)** | **.05 (.04-.06)** |
| SRMR | **.04** | **.04** | **.06** |
| **3. Body Fat (%)** | | | |
| Chi-square (df) | **21.61(21), p=.42** | 67.03(35), p<.001 | 148.82(75), p<.001 |
| CFI/TLI | **.99/.99** | **.96/.93** | **.93/.90** |
| RMSEA (90% CI) | **.01 (.00-.04)** | **.05 (.03-.07)** | **.05 (.04-.06)** |
| SRMR | **.04** | **.05** | **.07** |
| **4. COVID recruitment status** | | | |
| Chi-square (df) | 35.22(21), p<.05 | 72.44(35), p<.001 | 172.99(75), p<.001 |
| CFI/TLI | **.95/.92** | **.93**/.89 | .88/.83 |
| RMSEA (90% CI) | **.04 (.01-.06)** | **.05 (.04-.07)** | .06 **(.05-.07)** |
| SRMR | **.04** | **.05** | **.07** |
| **5. Hormonal contraceptive use** | | | |
| Chi-square (df) | **29.76(21), p=.10** | 85.51(35), p<.001 | 175.36(75), p<.001 |
| CFI/TLI | **.97/.95** | **.90**/.85 | .88/.83 |
| RMSEA (90% CI) | **.03 (.00-.06)** | .06 **(.04-.08)** | .06 **(.05-.07)** |
| SRMR | **.04** | **.05** | **.07** |
| **6. Mood disorders (current)** | | | |
| Chi-square (df) | **26.68(21), p=.18** | 75.52(35), p<.001 | 168.69(75), p<.001 |
| CFI/TLI | **.98/.97** | **.92**/.88 | .89/.85 |
| RMSEA (90% CI) | **.03 (.00-.05)** | **.05 (.04-.07)** | .06 **(.05-.07)** |
| SRMR | **.04** | **.05** | **.07** |
| **7. Selective serotonin reuptake inhibitor use** | | | |
| Chi-square (df) | **27.51(21), p=.15** | 76.34(35), p<.001 | 166.23(75), p<.001 |
| CFI/TLI | **.98/.96** | **.92**/.87 | .89/.85 |
| RMSEA (90% CI) | **.03 (.00-.05)** | **.05 (.04-.07)** | .06 **(.04-.07)** |
| SRMR | **.04** | **.05** | **.07** |
| **8. Substance use disorder (current)** | | | |
| Chi-square (df) | **25.94(21), p=.21** | 74.49(35), p<.001 | 164.79(75), p<.001 |
| CFI/TLI | **.98/.97** | **.93**/.88 | .89/.85 |
| RMSEA (90% CI) | **.02 (.00-.05)** | **.05 (.04-.07)** | **.06 (.04-.07)** |
| SRMR | **.04** | **.05** | **.07** |

Abbreviations: ^a^participant recruitment either before (0) or after (1) the COVID-19 pandemic onset; BMI=body mass index; CFI=comparative fit index; CI= confidence interval; df=degrees of freedom; RMSEA= root mean square error of approximation; TLI=Tucker-Lewis Index; SRMR= standardized root mean square residual.

Notes: Bolded font indicates values that meet threshold for good fit. Covariates included in rows 4-8 represent binary variables coded as 0 (no/not present) or 1 (yes/present).

**Table S3**. Sensitivity Analyses: Comparison of non-nested models using Akaike Information Criterion (AIC) and the Bayesian Information Criterion (BIC)

|  | **AIC** | **BIC** | **Sample size adjusted-BIC** | **Δ in BIC from model without covariate^a^** |
| --- | --- | --- | --- | --- |
| **Model 1**  **Eating/ Binge Eating Episode size**  **(no covariates)** | 8560.30 | 8652.04 | 8579.06 |  |
| Age | 8564.68 | 8676.37 | 8587.52 | -24.33 |
| BMI | 8292.07 | 8403.76 | 8314.91 | **248.28** |
| Body Fat | 8323.12 | 8434.81 | 8345.97 | **217.23** |
| COVID | 8555.42 | 8667.11 | 8578.26 | -15.07 |
| HC | 8565.24 | 8676.93 | 8588.09 | -24.89 |
| Mood dx | 8542.08 | 8653.77 | 8564.92 | -1.73 |
| SSRIs | 8566.12 | 8677.81 | 8588.96 | -25.77 |
| SUD | 8558.59 | 8670.28 | 8581.44 | -18.24 |
| **Model 2**  **LOC Frequency**  **(no covariates)** | 12908.13 | 13035.77 | 12934.24 |  |
| Age | 12912.51 | 13060.10 | 12942.70 | -24.33 |
| BMI | 12642.52 | 12790.11 | 12672.71 | **245.66** |
| Body Fat | 12677.06 | 12824.65 | 12707.25 | **211.12** |
| COVID | 12908.27 | 13055.86 | 12938.46 | -20.09 |
| HC | 12897.16 | 13044.76 | 12927.35 | -8.99 |
| Mood dx | 12887.87 | 13035.46 | 12918.06 | 0.31 |
| SSRIs | 12909.95 | 13057.55 | 12940.14 | -21.78 |
| SUD | 12886.50 | 13033.74 | 12916.34 | 2.03 |
| **Model 3**  **EDE (no covariates)** | 15821.67 | 16013.14 | 15860.84 |  |
| Age | 15828.12 | 16047.51 | 15872.99 | -34.37 |
| BMI | 15524.56 | 15743.95 | 15569.44 | **269.19** |
| Body Fat | 15565.60 | 15784.99 | 15610.47 | **228.15** |
| COVID | 15821.97 | 16041.36 | 15866.84 | -28.22 |
| HC | 15814.41 | 16033.80 | 15859.29 | -20.66 |
| Mood dx | 15768.31 | 15987.70 | 15813.18 | **25.44** |
| SSRIs | 15820.88 | 16040.28 | 15865.76 | -27.14 |
| SUD | 15802.09 | 16021.48 | 15846.97 | -8.34 |

Abbreviations: ^a^ Higher change score values favor model with covariate; values <10 favor original (more parsimonious) model; AIC= Akaike Information Criterion; BIC= Bayesian Information Criterion; BMI= body mass index; EDE= eating disorder examination global score; LOC= loss of control eating

**Table S4.** Sensitivity Analyses: Fit Statistics and Standardized Estimates for Structural Equation Models with Body Mass Index as a Covariate

|  | **Outcome** | | |
| --- | --- | --- | --- |
|  | **Model 1b**  **Eating/Binge Eating Episode Size** | **Model 2b**  **Loss of Control Frequency** | **Model 3b**  **Global Eating Disorder Severity** |
| **Fit Statistics (threshold)** |  |  |  |
| Chi-square (df) (p>.01) | **22.76(21), p=.36** | 64.96(35), p<.001 | 144.74(75), p<.001 |
| CFI/TLI (≥ 0.90) | **0.99/0.99** | **0.96/0.94** | **0.94/0.91** |
| RMSEA [90% CI] (≤0.05; [.0.00, ≤0.08]) | **0.01 [0.00, 0.05]** | **0.05 [0.03, 0.06]** | **0.05 [0.04, 0.06]** |
| SRMR (≤0.08) | **0.04** | **0.04** | **0.06** |
| **Direct Paths: Standardized Estimates [95% CIs]** |  |  |  |
| WS to Leptin | **-0.15 [-0.22, -0.08]**^a^ | **-0.15 [-0.22, -0.08]**^a^ | **-0.15 [-0.22, -0.08]**^a^ |
| Leptin to GLP1 | **0.20 [0.05, 0.36]**^a^ | **0.20 [0.05, 0.36]**^a^ | **0.20 [0.05, 0.36]**^a^ |
| GLP1 to satiation (behavior) | **-0.17 [-0.29, -0.03]**^a^ | NA | **-0.17 [-0.29, -0.03]**^a^ |
| GLP1 to satiation (self-report) | **0.12 [0.01, 0.23]**^a^ | NA | **0.12 [0.01, 0.23]**^a^ |
| GLP1 to RV-E (behavior) | NA | -0.03 [-0.17, 0.12] | -0.03 [-0.16, 0.12] |
| GLP1 to reward valuation (self-report) | NA | 0.02 [-0.14, 0.20] | 0.03 [-0.14, 0.20] |
| Satiation (behavior) to outcome | 0.12 [-0.02, 0.25] | NA | 0.06 [-0.05, 0.18]^a^ |
| Satiation (self-report) to outcome | -0.06 [-0.17, 0.05] | NA | **0.12 [0.01, 0.23]**^a^ |
| RV-E (behavior) to outcome | NA | -0.05 [-0.22, 0.11] | <0.01 [-0.15, 0.16] |
| Reward valuation (self report) to outcome | NA | 0.09 [-0.06, 0.26] | <-0.01 [-0.15, 0.15] |
| BMI to Leptin | **0.71 [0.66, 0.76]^a^** | **0.71 [0.66, 0.76]^a^** | **0.71 [0.66, 0.76]^a^** |
| BMI to GLP1 | -0.05 [-0.20, 0.11] | -0.05 [-0.21, 0.10] | -0.05 [-0.21, 0.10] |
| BMI to satiation (behavior) | **0.21 [0.11, 0.32]^a^** | NA | **0.23 [0.12, 0.33]^a^** |
| BMI to satiation (self-report) | -0.03 [-0.14, 0.09] | NA | -0.04 [-0.15, 0.08] |
| BMI to RV-E (behavior) | NA | **0.18 [0.04, 0.31]^a^** | **0.18 [0.05, 0.31]^a^** |
| BMI to reward valuation (self-report) | NA | 0.08 [-0.06, 0.21] | 0.08 [-0.06, 0.21] |
| BMI to outcome | **0.15 [0.05, 0.24]^a^** | **0.18 [0.07, 0.28]^a^** | **0.29 [0.21, 0.37]^a^** |
| **Indirect Path: 95% CIs** |  |  |  |
| WS to outcome via satiation (behavior) | **>0.000, 0.002** | NA | **>0.000, 0.001** |
| WS to outcome via satiation (self-report) | **>0.000, 0.001** | NA | -0.002, 0.000 |
| WS to outcome via RV-E (behavior) | NA | -0.001, 0.000 | 0.000, 0.000 |
| WS to outcome via reward valuation (self-report) | NA | -0.001, 0.001 | **>0.000, 0.001** |

Table S4 Notes: Bolded font indicates values that meet threshold (fit statistics) or statistically significant direct pathways and indirect pathways that do not cross zero.

Table S4 Abbreviations: ^a^*p*<0.05; CFI=comparative fit index; CIs=Confidence Intervals; df= degrees of freedom; GLP1=Glucagon-like peptide 1 response (total); RMSEA= root mean square error of approximation; RV=reward valuation; RV-E=Reward valuation-effort; SRMR= standardized root mean square residual; TLI=Tucker-Lewis Index; WS=weight suppression. Bold font denotes values that achieve threshold for good fit; RV-E=Reward valuation-effort; WS=weight suppression.

**Table S5.** Comparison between Bulimia Nervosa-Spectrum (BN-S) and Control Participants on Study Variables.

|  |  | **Controls**  (N range=43-78) | **BN-S**  (N range=228-321) |  |  |  |
| --- | --- | --- | --- | --- | --- | --- |
|  |  | **Mean (SD)** | **Mean (SD)** | ***t* (df)** | ***p*** | **Cohen’s *d*** |
|  | 1. Weight Suppression (WS), % | 4.12 (4.09) | 6.96 (6.58) | -4.80(186.39)^a^ | **<0.01** | 0.46 |
|  | 2. Leptin, ng/mL | 19.95 (17.64) | 31.91 (21.93) | 22.77 (1)^b^ | **<0.01** | 0.06^c^ |
|  | 3. Glucagon-like peptide 1 (GLP-1) total AUC, pM x min | 1325.57 (682.20) | 1390.91 (608.55) | 0.03 (1)^b^ | 0.85 | <0.01^c^ |
|  | 4. Ad Lib Intake, grams | 201.75 (112.38) | 258.75 (155.05) | -2.74 (285) | **<0.01** | 0.39 |
|  | 5. Visual Analogue Scale (VAS) hunger (reverse scored), AUC, mm x min | 2796.38 (765.34) | 3024.12 (760.30) | -2.13 (296) | **<0.01** | 0.30 |
|  | 6. VAS fullness, AUC, mm x min | 2344.85 (718.16) | 2382.36 (780.06) | -0.35 (296) | 0.73 | 0.05 |
|  | 7. VAS satiation, AUC, mm x min | 1920.00 (887.53) | 1946.45 (827.41) | -0.22 (295) | 0.82 | 0.03 |
|  | 8. Reward Valuation – Effort (RV-E) food reward, breakpoint | 608.14 (413.02) | 800.00 (474.92) | 5722.50 (436.02)^d^ | **<0.01** | 0.02^c^ |
|  | 9. RV-E game reward in fasted state, breakpoint | 747.22 (478.25) | 870.36 (476.17) | 11645.00 (748.94)^d^ | **0.02** | 0.03^c^ |
|  | 10. RV-E game in fed state, breakpoint | 602.38 (440.99) | 611.40 (397.24) | 7385.00 (580.66)^d^ | 0.73 | <0.01^c^ |
|  | 11. VAS want food reward before task, mm | 62.81 (28.54) | 67.15 (27.46) | -0.94 (253) | 0.35 | 0.16 |
|  | 12. VAS want game reward before fasted task, mm | 44.51 (24.63) | 46.28 (23.38) | -0.56 (342) | 0.57 | 0.08 |
|  | 13. VAS want game reward before fed task, mm | 36.16 (26.18) | 34.21 (27.25) | 0.51 (292) | 0.61 | 0.07 |
|  | 14. Eating/Binge eating episode size, kcal | 1214.76 (496.13) | 2975.11 (1548.90) | -17.07 (371.80)^a^ | **<0.01** | 1.25 |
|  | 15. Loss of control (LOC) frequency, no. in 12 weeks | NA | 43.65 (40.51) | NA | NA | NA |
|  | 16. Eating Disorder Examination (EDE) global score | 0.09 (0.12) | 3.17 (0.96) | -55.94 (355.22)^a^ | **<0.01** | 3.58 |
|  | 17. Age, yrs | 20.41 (2.75) | 20.22 (2.54) | 0.58 (397) | 0.57 | 0.07 |
|  | 18. Body Mass Index (BMI), kg/m2 | 22.22 (3.20) | 25.06 (4.29) | -6.55 (152.33)^a^ | **<0.01** | 0.69 |
|  | 19. Percent Body Fat, % | 26.58 (7.29) | 31.79 (7.79) | -5.37 (397) | **<0.01** | 0.68 |
|  |  | **% Yes** | **% Yes** | **χ^2^(1)** | ***p*** | **ϕ** |
|  | 20. Hormonal Contraceptive (HC) Use (0=no; 1=yes) | 47.4% | 45.5% | 0.10 | 0.76 | 0.02 |
|  | 21. Selective Serotonin Reuptake Inhibitor (SSRI) Use (0=no; 1=yes) | 5.1% | 10.6% | 2.17 | 0.14 | 0.07 |
|  | 22. Current DSM-5 depressive disorder diagnosis (0=no; 1=yes) | 3.9% | 36.8% | 32.17 | **<0.01** | 0.28 |
|  | 23. Current DSM-5 substance use disorder diagnosis (0=no; 1=yes) | 10.3% | 39.9% | 24.50 | **<0.01** | 0.25 |
|  | 24. Enrolled after COVID-19 pandemic onset (0=no; 1=yes) | 42.3% | 20.3% | 16.48 | **<0.01** | 0.20 |

^a^Levene’s test for equality of variances significant at p<.001, used t-test for equal variances not assumed; ^b^F statistic, ANOVA controlling for assay kit in which plasma samples were run; ^c^eta-squared; ^d^Mann-Whitney U test for ordinal data with (standard error).

**Table S6.** Fit Statistics for Structural Equation Models (SEMs) and Standardized Estimates with Bootstrapped 95% Confidence Intervals for Paths in SEMs in Participants with an Eating Disorder (Bulimia Nervosa-Spectrum; N=321).

|  | **Outcome** | | |
| --- | --- | --- | --- |
|  | **Model 1**  **Eating/Binge Eating Episode Size** | **Model 2**  **Loss of Control Frequency** | **Model 3**  **Global Eating Disorder Severity** |
| **Fit Statistics (threshold)** |  |  |  |
| Chi-square (df) (p>.01) | **12.86 (19), p=.85** | **43.42(31), p=.07** | 122.42(69), p<.001 |
| CFI/TLI (≥ 0.90) | **1.00/1.00** | **0.97/0.95** | **0.91**/0.88 |
| RMSEA [90% CI] (≤0.05; [.0.00, ≤0.08]) | **0.00 [0.00, 0.03]** | **0.04** **[0.00, 0.06]** | **0.05** **[0.04, 0.06]** |
| SRMR (≤0.08) | **0.03** | **0.05** | **0.08** |
| **Direct Paths: Standardized Estimates [95% CIs]** |  |  |  |
| WS to Leptin | **-0.28 [-0.37, -0.18]^a^** | **-0.28 [-0.37, -0.19]^a^** | **-0.28 [-0.37, -0.18]^a^** |
| Leptin to GLP1 | **0.22 [0.09, 0.34]^a^** | **0.21 [0.08, 0.34]^a^** | **0.21 [0.08, 0.34]^a^** |
| GLP1 to satiation (behavior) | *-0.14 [-0.29, 0.01*]^b^ | NA | *-0.14* *[-0.29, 0.01]^b^* |
| GLP1 to satiation (self-report) | **0.15 [0.03, 0.26]^a^** | NA | **0.15 [0.02, 0.26]^a^** |
| GLP1 to RV-E (behavior) | NA | -0.04 [-0.19, 0.12] | -0.04 [-0.20, 0.12] |
| GLP1 to RV (self report) | NA | 0.06 [-0.14, 0.25] | 0.07 [-0.13, 0.25] |
| Satiation (behavior) to outcome | 0.10 [-0.06, 0.25] | NA | 0.03 [-0.12, 0.16] |
| Satiation (self-report) to outcome | -0.08 [-0.21, 0.04] | NA | **0.17 [0.03, 0.30]^a^** |
| RV-E (behavior) to outcome | NA | -0.14 [-0.34, 0.05] | -0.03 [-0.22, 0.16] |
| RV (self report) to outcome | NA | *0.17 [-0.03, 0.37]^b^* | 0.04 [-0.15, 0.23] |
| **Indirect Path: 95% CIs** |  |  |  |
| WS to outcome via satiation (behavior) | -0.001, 0.004 | NA | -0.001, 0.002 |
| WS to outcome via satiation (self-report) | **>0.000, 0.003** | NA | -0.004, >0.000 |
| WS to outcome via RV-E (behavior) | NA | -0.002, 0.002 | -0.001, 0.001 |
| WS to outcome via RV (self-report) | NA | -0.003, 0.002 | -0.001, 0.002 |

^a^*p*<0.05. ^b^*p*<0.10. Notes: Bold font denotes values that achieve threshold for good fit or statistically significant direct pathways and indirect pathways that do not cross zero. Italics denotes values that approach traditional threshold for statistical significant and achieve statistical significance based on a one-tailed test of *a priori* hypotheses. Abbreviations: CFI=comparative fit index; CIs=Confidence Intervals; GLP1=Glucagon-like peptide 1 response (total); RMSEA= root mean square error of approximation; RV=reward valuation; RV-E=Reward valuation-effort; SRMR= standardized root mean square residual; TLI=Tucker-Lewis Index; WS=weight suppression.
